# Decoding the Genetic Alterations in Genes of *DNMT* Family (DNA Methyl-Transferase) and their Association with Head and Neck Squamous Cell Carcinoma

**DOI:** 10.31557/APJCP.2020.21.12.3605

**Published:** 2020-12

**Authors:** Tahreem Fathima, Paramasivam Arumugam, Smiline Girija AS, J Vijayashree Priyadharsini

**Affiliations:** 1 *Saveetha Dental College, Velappanchavadi, Poonamallee High Road, India. *; 2 *Dental Research Cell, Saveetha Dental College, Poonamallee High Road, Chennai-77, India. *; 3 *Department of Microbiology, Saveetha Dental College, Saveetha Institute of Medical and Technical Sciences (SIMATS), Saveetha University, Chennai, India. *; 4 *Biomedical Research Unit and Laboratory Animal Centre - Dental Research Cell, Saveetha Dental College and Hospitals, Chennai-77, India. *

**Keywords:** HNSCC, in silico, methyltransferases, mutations

## Abstract

**Objective:**

Epigenetic modifications are gaining focus due to their indirect association with tumorigenesis. DNA methylation plays a prime role in regulation of gene expression. Any aberrations in this gene family may lead to chromosomal instability and increased magnitude of tumour progression. In line with the above fact, the present study has been designed to identify genetic alterations in the genes of the DNMT (DNA methyl-transferase) family among head and neck squamous cell carcinoma patients (HNSCC).

**Methods:**

The present study follows an observational design employing computational tools for analysis. The TCGA-Firehose Legacy data was assessed using the cBioportal database. The dataset comprised of 530 samples from HNSCC patients which were assessed for genetic alterations in the DNMT family. Furthermore, the protein stability analysis and pathogenicity of the mutations were assessed using I-Mutant Suite and PROVEAN tools.

**Results:**

Almost all genes of the *DNMT* family harboured gene amplification. The *TRDMT1* and *DNMT3L* genes showed deep deletions. Apart from these several non-synonymous, truncating and splice-site mutations were also documented. Protein stability and pathogenicity analysis revealed that majority of the mutations were found to decrease the stability and impose pathogenicity. Upon probing for reported mutations using gnomAD database, around six reference single nucleotide polymorphisms were identified which were found to exhibit a minor allele frequency less than 0.01.

**Conclusions:**

Screening of an exhaustive collection of patient’s samples could provide immense knowledge about the disease pathogenesis and identification of therapeutic leads. The variants identified in the present study could be used as diagnostic markers. However, further experimental analysis through genotyping assay is warranted to validate the present findings.

## Introduction

Head and neck squamous cell carcinoma (HNSCC) is the sixth most common type of cancer which is responsible for over 350,000 deaths every year (McDermott and Bowles, 2019). According to the GLOBOCAN report 2012, cancer of lip and oral cavity was found to be the 12th most common cancer in Asia (Gupta et al., 2016). Recent reports by the global cancer observatory, GLOBOCAN, 2018, revealed an increase in the mortality rate due to cancer of the lip and oral cavity especially in males, with a significant proportion of cases arising from south Asian countries (Bray et al., 2018). These two reports have projected a steep increase in the incidence of the disease. The risk for oral cancer is precipitated by several factors such as tobacco chewing, chronic alcoholism, smoking, HPV (Human Papilloma Virus) infection, sharp tooth in addition to the genetic makeup (Ram et al., 2011). The incidence of HNSCC is high in males when compared to females, especially in eastern Europe and India with over 20 males affected per 100, 000 individuals (Fitzmaurice et al., 2017). Smoking tobacco such as cigarettes and pipes along with smokeless tobacco increased the risk of HNSCC by 2-4 fold (Wyss et al., 2013, Zhou et al., 2013). The practice of betel nut chewing, a habit most commonly seen in the Asian subcontinent increased the risk of HNSCC from 2 - 15 fold (Guha et al., 2014). 

Epigenetic alterations in cancer have gained a lot of attention in the recent years. Genetic alterations spanning the genes encoding the enzymes methyltransferases have been shown to be associated with numerous cancers and pre-cancerous lesions. The process of methylation inactivates crucial genes without interfering with the sequence. Hypermethylation of promoter regions of cell cycle control genes, tumor suppressor genes are known to diminish the function, thereby contributing to the malignant transformation of the cells (Rotondo et al., 2018; Casalino and Verde, 2020). An in-depth knowledge on the expression profile of genes coding for methyltransferases under normal and pathological conditions would aid in identifying the triggers associated with these epigenetic marks. In line with the above facts, DNMTs are known to play a crucial role in the process of tumorigenesis. Defects in DNMTs is accompanied by chromosomal imbalances which results in the remodelling of chromatin, instability of genome and inactivation of genes. Although global hypomethylation is common with tumor cells, hypermethylation of specific regions have resulted in silencing of crucial genes involved in the repair or cell cycle regulatory pathways (Rodriguez-Paredes and Esteller, 2011). There are five *DNMTs* discovered in the human genome till date viz., *DNMT1, DNMT2, DNMT3A, DNMT3B* and *DNMT3L*. The *DNMT1, DNMT3A* and *DNMT3B* are canonical enzymes which take part in the catalytic activity, whereas the other *DNMTs*, i.e., *DNMT2 *and *DNMT3L* are non-canonical family members as they do not possess the catalytic activity (Lyko, 2019). A few genes known to be methylated or otherwise silenced in oral premalignant lesions are *p16, MGMT, RAR-beta -2, cadherin* and *DAP kinase *(Díez-Pérez et al., 2011). Other genes have been found to be hypermethylated in oral cancer, such as* ZNF582, PAX1* (Cheng et al., 2018; Cheng et al., 2016) and *EYA4* (Towle et al., 2016). The DNMT inhibitors have been tested to assess their efficacy on animal models. One such experiment included the DNMT inhibitor, 5-Aza-2’-deoxycytidine in combination with a low dose of trans-retinoic acid on a murine model of oral carcinoma, induced using the carcinogen, 4-nitroquinoline 1-oxide (4-NQO). The results revealed that the combination was effective in attenuating tongue lesion severity when compared to the other treatment modalities used for the purpose. Hence, a combination of DNA demethylating drugs could serve as a promising strategy to reduce cancer of oral cavity (Tang et al., 2009).

Several studies have identified polymorphic variants in *DNMT *genes with both positive and negative associations with different cancer types. Promoter polymorphisms v*iz.,-149C>T, -283T>C,* and *-579G>T* located in the DNMT3B gene did not produce any significant association with nasopharyngeal carcinoma in Taiwanese population (Chang et al., 2008). A meta-analysis conducted by Zhu et al., (2015) suggested that the risk of head and neck cancer was associated with heterozygous variant of DNMT3B which is -149C/T. Duan et al., demonstrated that the variants (rs2424913 C/T, rs1569686 G/T, rs6087990 T/C and rs2424908 T/C) were either negatively associated with cancer risk or confer protection against cancer in Asian population.

## Materials and Methods

*Sample data set*

The cBioPortal for Cancer Genomics (http://cbioportal.org) integrates an exhaustive collection of molecular profiling information from cancer tissues and cell lines (Cerami et al., 2012; Gao et al., 2013). The database is user friendly and hosts genetic, epigenetic and proteomic information of the cases registered. The sample data set includes 528 HNSCC cases (530 samples) of which 504 samples harboured information about copy number variations and mutation data. The demographic details of cases in the head and neck squamous cell carcinoma (TCGA, Firehose Legacy) dataset were recorded (Table 1). 

*Oncoprint data analysis*

Submission of user defined query of 5 genes belonging to the family of DNA methyltransferase (Data not shown) returned a window with OncoPrint data which demonstrated the presence of alterations in 5 crucial genes of the *DNMT* family viz., *DNMT1, TRDMT1, DNMT3A and DNMT3B, DNMT3L. *The somatic mutation frequency and the site of mutation in the candidate genes were documented (Cerami et al., 2012; Gao et al., 2013) (Table 2). 

*gnomAD analysis*

The genome aggregation database (gnomAD) hosts a comprehensive information of data spanning 125,748 exome sequences and 15,708 whole genome sequences from unrelated individuals sequenced and deposited as part of various disease-specific or population genetic studies. A subtractive analysis was carried out to identify those variants which were not previously reported. Hence, such variants were designated as novel variants. The search also provided an insight about the minor allele frequency of the variants in the population by which the nature of the variants can be ascertained (Karczewski et al., Version 4, 2020) (Table 2).

*Protein stability analysis*

The I-Mutant server was used for prediction of protein stability changes upon single nucleotide mutations leading to change in the amino acid being encoded by the triplet codon. The server uses either protein sequence or structure to predict stabilisation and destabilisation of protein structure in majority of cases. The protein sequence coded by the genes were downloaded in the FASTA format from the public domain (https://www.ncbi.nlm.nih.gov/protein/). These sequences were further analysed upon substitution with the variant amino acid. The changes in the stability of the proteins were assessed using the free energy stability change (ΔΔG) value. A value greater than zero implies increase in protein stability and a value less than zero is considered to decrease the protein stability (Capriotti et al., 2005) (Table 3).

*Pathogenicity analysis*

PROVEAN (Protein Variation Effect Analyzer) predicts the consequences of an amino acid substitution in a protein during a mutational event (Table 3). The present analysis employs a user defined query of missense variants identified in a specific gene entered along with the reference sequence of the protein derived from the NCBI database. The default cut-off value of -2.5 was set prior to analysis. Based on the scores the amino acid substitutions are classified as either neutral or deleterious (Choi and Chan, 2015). A score < -2.5 or > -2.5 was considered to be deleterious and neutral respectively (Table 3).

*Gene expression analysis*

Gene expression analysis on the *DNMT1, TRDMT1, DNMT3A, DNMT3B* and *DNMT3L* genes were carried out using the UALCAN database (http://ualcan.path.uab.edu/cgi-bin/TCGA- survival1.pl?genenam) (Figure 2). Expression data was represented as transcripts per million (TPM) which is a normalization method for RNA- seq data. Further, these TPM values were used for the generation of box- whisker plots which was employed to determine the significant difference between the groups. Survival effect analysis of gene expression were assessed using multivariate Kaplan - Meier survival analysis (Chandrasekar et al., 2017). 

## Results

*Demographic data*

The dataset (TCGA, Firehose Legacy) included in the present study had information on 528 HNSCC patients (530 samples) of which mutations and copy number variation data was available for 504 samples. The male:female ratio was found to be 2.7:1, with age group ranging from 19 - 90 years. The number of individuals with the history of smoking and alcohol was roughly around 98% and 67%. The dataset had samples from patients of American (85.6%), African (9.1%), Asian (2.1%) and American Indian (0.4%) decent. The distribution of patients based on the histologic grade of neoplasm is given in Table 1, of which 59% of patients had grade 2 tumor.

*Oncoprint data analysis*

**Figure 1:**
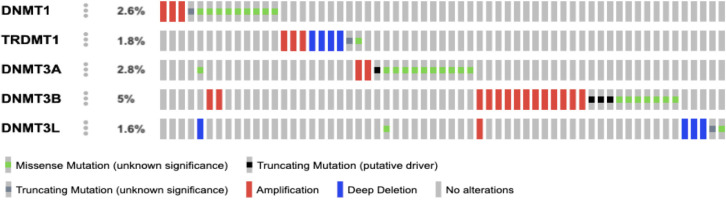
The Oncoprint Data Depicts the Gene Alterations in DNMT Family of Genes. Each of the grey bar represents HNSC patients. The colour codes represents genetic alterations in individual patient

The oncoprint data analysis revealed gene amplification in all five genes viz., *DNMT1, TRDMT1, DNMT3A,* *DNMT3B* and *DNMT3L*, of which* DNMT3B* was found to harbour highest frequency of gene amplification. The genes *TRDMT1* and *DNMT3L* demonstrated deep deletions, whereas gene amplification was observed in all the genes investigated. The *DNMT3B *gene possessed the highest frequency of variations/mutations (5%) from among all the genes identified with alterations (Table 2). Several truncating and mis-sense variants of unknown significance have been documented (Figure 1). Truncating mutations identified in *DNMT3A* gene (E371*) and *DNMT3B* gene (C496=, K441Efs*11, R576*) were found to be putative drivers. The clinical implication states that they are likely oncogenic with a biological effect involving loss of function. Novel variants were common among the genes* DNMT1* and *DNMT3L. *

*gnomAD analysis*

A total of 6 reported variants were identified using gnomAD analysis viz., rs200204830 in *TRDMT1*, rs757211277, rs566390868, rs769590067 in DNMT3A and rs121908946, rs770751820 in *DNMT3B* genes. All the variants identified in the present study had a minor allele frequency < 0.01, implying the fact that these rare variants might be associated with risk of a particular disease (Table 2).

*Protein stability and pathogenicity analysis*

Stability of the protein largely affects the biological function of the protein. Hence, protein stability was assessed for all the non-synonymous variants identified in the study. Majority of mis-sense variants observed were found to decrease the stability of the protein product, thereby giving away a chance for influencing the catalysis process. A few of the non-synonymous mutations in *DNMT3A* gene showed an increase in stability. Although, presented with decreased stability all the variants were not found to lead to a deleterious phenotype. Majority of the variants produced deleterious effect with exception in a few gene variants exhibiting neutral outcomes viz., *TRDMT1* and *DNMT3L* (Table 3). 

*Gene expression analysis*

**Figure 2:**
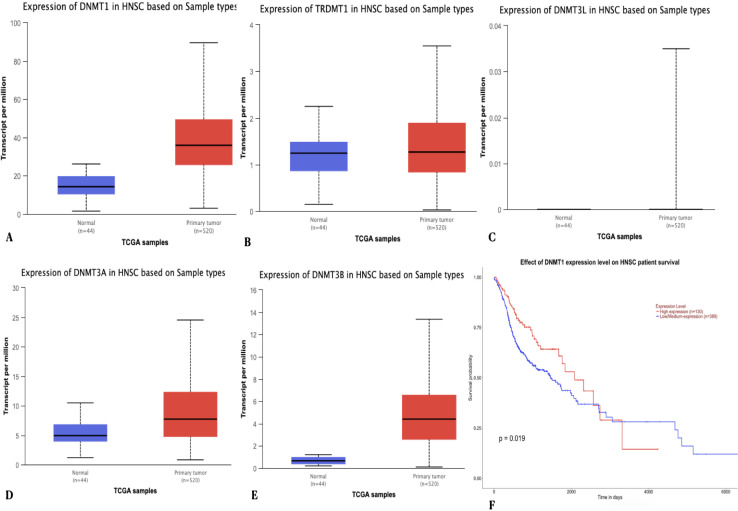
The Box Whisker Plot and Kaplan Meier Survival Analysis Demonstrating Differential Gene Expression Pattern and Survival Probability of HNSCC Patients Respectively. Upregulation of genes (A) *DNMT1 *(B) *TRDMT1 *(C) *DNMT3A* (D)* DNMT3B* and (E) *DNMT3L* when compared to normal samples. (F) The survival plot showed that the low/medium level expression of *DNMT1* gene resulted in lower survival probability when compared to patients with high level expression

The gene expression data revealed that almost all the genes in the study panel presented with a statistically significant differential gene expression pattern (Figures 2A - 2E). The p values for each of the gene as computed is as follows: *DNMT1* (<1 x 10^-12^), *TRDMT1* (7.64 x 10^-4^), *DNMT3A* (1.62 x 10^-12^), *DNMT3B* (<1 x 10^-12^) and *DNMT3L* (2.22 x 10^-3^). The Kaplan-Meier survival analysis for *DNMT1* gene expression returned a significant result between the patients with high level and low/medium level expression (p value = 0.019). The patients with low/medium level gene expression showed a decreased survival probability when compared to patients with high level expression of *DNMT1*. A p value less than 0.05 was considered to be significant (Figure 2F).

**Table 1:** Demographic Details of Patients Analyzed in the Present Study (as Obtained from the cBioportal Site)

Variable	Number
Gender	
Male	386
Female	142
Mutation count	6-3181
Diagnosis age	19-90 years
Smoking status	
Smokers	515
Data not available	12
Unknown	1
Alcohol history	
Yes	352
No	165
Data not available	11
Neoplasm Histologic grade	
Grade 1	63
Grade 2	311
Grade 3	125
Grade 4	7
Grade GX	18
Data not available	4
Race category	
White	452
African	48
Asian	11
American Indian or Alaska native	2
Data not available	15

**Table 2:** Description on the Genes, Protein Encoded, Genetic Alterations, Loci, Frequency of Variant Allele in Tumor Sample in DNA Methyltransferase Family of Genes

Gene	Protein	Alteration	Loci	% of alteration	Variant allele frequency in tumor sample	gnomAD frequency data
*DNMT1*	DNA methyltrasferase 1	Gene amplification	19q13.2	2.6		
		E559Q			0.03	Novel
		P1330S			0.17	Novel
		P1325S			0.2	Novel
		E912Q			0.05	Novel
		S1352G			0.2	Novel
		P692S			0.21	Novel
		H370Y			0.03	Novel
		T616M			0.42	Novel
		R325L			0.09	Novel
		X891_splice			0.05	Novel
		P1106H			0.04	Novel
*TRDMT1* *(DNMT2)*	TRNA Aspartic Acid Methyltransferase 1	Gene amplification	10p13	1.8		
		Deep deletions				
		A198E			0.44	rs200204830
		S272*			0.22	Novel
*DNMT3A*	DNA methyltrasferase 3 alpha	Gene amplification	2p23.3	2.8		
		E371*			0.31	Novel
		R729Q			0.52	rs757211277
		E491D			0.12	Novel
		S689C			0.17	Novel
		R556M			0.16	Novel
		R488Q			0.13	rs566390868
		Q374L			0.16	Novel
		P419L			0.12	Novel
		P106S			0.08	Novel
		G150V			0.12	Novel
		W893R			0.45	Novel
		E907K			0.14	rs769590067
		R899C			0.18	Novel
*DNMT3B*	DNA methyltrasferase 3 beta	Gene amplification	20q11.21	5		
		R576*			0.14	Novel
		K441Efs*11			0.41	Novel
		C496=			0.05	Novel
		Q328E			0.26	Novel
		E464Q			0.16	Novel
		R840Q			0.25	rs121908946
		R485L			0.12	Novel
		P845S			0.22	Novel
		H132R			0.29	rs770751820
		Q196H			0.42	Novel
*DNMT3L*	DNA methyltrasferase 3 like	Gene amplification	21q22.3	1.6		
		Deep deletions				
		R47*			0.13	Novel
		P310S			0.22	Novel
		S325N			0.21	Novel

**Table 3:** Consequences of Non-Synonymous Variations on Protein Stability and Pathogenicity as Assessed by IMutant and PROVEAN Tools

Gene	Variation	I-Mutant prediction	Score	PROVEAN Score	Prediction
*DNMT1*	E559Q	Decrease	-0.33	-2.024	Neutral
	P1330S	Decrease	-1.09	-6.84	Deleterious
	P1325S	Decrease	-1.47	-6.973	Deleterious
	E912Q	Decrease	-0.67	-1.683	Neutral
	S1352G	Decrease	-3.01	-2.551	Deleterious
	P692S	Decrease	-0.11	-7.006	Deleterious
	H370Y	Decrease	-0.11	-2.633	Deleterious
	T616M	Decrease	-0.84	-4.531	Deleterious
	R325L	Decrease	-0.13	-0.594	Neutral
	P1106H	Decrease	-0.25	-1.505	Neutral
*TRDMT1 (DNMT2)*	A198E	Decrease	-1.22	-0.585	Neutral
*DNMT3A*	R729Q	Decrease	-0.21	-3.695	Deleterious
	E491D	Increase	0.05	-2.742	Deleterious
	S689C	Decrease	-1.25	-3.432	Deleterious
	R556M	Increase	0.97	-5.288	Deleterious
	R488Q	Decrease	-1.39	-1.513	Neutral
	Q374L	Increase	0.74	-4.303	Deleterious
	P419L	Increase	0.38	-2.962	Deleterious
	P106S	Increase	0.03	-1.025	Neutral
	G150V	Decrease	-1.61	-0.38	Neutral
	W893R	Decrease	-1.36	-13.22	Deleterious
	E907K	Decrease	-0.7	-3.13	Deleterious
	R899C	Decrease	-1.7	-7.55	Deleterious
*DNMT3B*	Q328E	Increase	0.94	-0.838	Neutral
	E464Q	Decrease	-0.95	-2.561	Deleterious
	R840Q	Decrease	-1.9	-3.531	Deleterious
	R485L	Decrease	-0.37	-3.598	Deleterious
	P845S	Decrease	-1.72	-7.328	Deleterious
	H132R	Decrease	-0.12	-0.699	Neutral
	Q196H	Decrease	-0.79	-1.144	Neutral
*DNMT3L*	P310S	Decrease	-1.62	0.389	Neutral
	S325N	Decrease	-0.62	-0.9	Neutral

## Discussion

The most important epigenetic marks which is considered to be the key players in gene silencing is DNA methylation (Zemach et al., 2010; Jeltsch and Gowher, 2019). The C5 (methylcytosine - 5mC), N6 (methyladenine - 6mA) methylations are known types of methylations identified in the eukaryotic genome. Similar catalytic mechanisms are exhibited by the family of DNA methyltransferases. The promoter methylation is usually encountered in the early stage of carcinogenesis process. A very recent study by Sánchez-Siles et al., (2019) investigated the association between T allele of the rs16906252 and O16-methylguanine-DNA methyltransferase (MGMT) with oral lichen planus. The results were convincing showing association between the genotype and the promoter region of *MGMT* gene. Since the process of DNA methylation is related to the silencing of genes, methylation of tumor suppressor genes may inevitably lead to uncontrolled cell division and accumulation of lesions in DNA. The present study has identified several novel variants which might have a profound effect on the clinical presentation of *HNSCC*. Variants previously reported viz., rs200204830 of *TRDMT1*, rs757211277, rs566390868, rs769590067 of* DNMT3A*, rs121908946, rs770751820 of *DNMT3B*. Protein stability and pathogenicity analysis revealed three variants *R729Q*, *E907K* (*TRDMT1*) and *R840Q* (*DNMT3B*) which were highly pathogenic with scores less than 3.0. 

The relationship between hypermethylation of C*DKN2A (p16)* and OSCC was reported in a study by Yakushiji et al., (2003) The *p16* gene was found to be hypermethylated in about 48% of the samples, with significant downregulation observed in both mRNA and protein expressions. Demethylation assay was also performed on OSCC derived cell lines which eventually resulted in the reactivation of *p16 *gene expression. The *DNMTs* over-expressed were *DNMT1, DNMT3A* and *DNMT3B* at 72%, 56% and 64% respectively. Although a definitive conclusion could not be drawn by the researchers about the connection between hypermethylation of p16 genes and up-regulation of *DNMTs*, they concluded by reporting the fact that both the processes are observed in oral carcinoma acting through different mechanisms. A similar kind of trend was observed in the present study, wherein an increase in gene expression was observed in all the genes selected. The differential gene expression observed was statistically significant for all the genes with *DNMT1, DNMT3A* and 3B showing a dramatic increase in the gene expression when compared to the normal group.

Gene expression and genotyping analysis performed from the samples obtained from OSCC patients revealed over-expression of *DNMT1* (36.9%), *DNMT3A* (26%) and *DNMT3B* (23%). The over-expression of *DNMT1 *was found to influence the overall survival of patients presenting with a p value of 0.003. Also, over-expression of *DNMT1* was considered to be an independent prognostic factor which showed a 2.385 times higher risk for the tumor to relapse in comparison to a lower expression level. Furthermore, the analysis of 2 polymorphisms in *DNMT1* (rs2228612) and *DNMT3B *(rs406193) by TaqMan SNPs genotyping assays demonstrated the fact that the former variant was associated with reduced survival of OSCC patients (p = 0.036). The present study has also identified several variants which led to protein de-stabilization exhibiting a deleterious consequences (Supic et al., 2017). The survival analysis of the HNSCC patients exhibiting a differential expression profile of the *DNMT1* gene exhibited a significant difference in the survival period. The patients with a high level expression of *DNMT1* had an increased survival rate in comparison to those with low/medium level expression. This fact was found to contradictory to the study conducted by Supic et al. Probing more into the genetic variations and influence of genetic and epigenetic factors could provide clues on the contrasting results obtained.

Chen et al., (2014) demonstrated through *in vitro *and *in vivo* experiments, that the inhibition of *DNMT3bB *resulted in slower progression of tumor, attenuated tumor invasion and epithelial mesenchymal transition. The activation of inflammatory marker IL-6 could be the vital signal responsible for the induction and over-expression of *DNMT3B* in oral cancer. In addition, the up-regulation of *DNMT3B* was significantly linked to the risk of lymph node involvement, recurrence of the disease and shorter survival period in patients in advanced stage of oral cancer. Hence, the researchers suggest that *DNMT3B *could be used as a promising therapeutic target for oral cancer. A recent study identified an anti-aging gene Klotho to be associated with carcinogenesis. The study group analyzed the association between methylation and the expression of Klotho which returned significant results indicating that the expression of *DNMT3*B was increased with a simultaneous reduction in the expression of Klotho gene in OSCC patients. Therefore it was concluded that downregulation of Klotho was associated with overexpression of *DNMT3B* in tumor tissues. As reported by several studies, DNMT3B was found to be the most commonly studied gene in relation to oral cancer. The present study also reported highest frequency of alteration in the* DNMT3B* gene when compared to the other members of the family. 

A very recent study by Shiah et al., (2020) demonstrated that epigenetic silencing of genes related retinoic acid signaling had a significant role to play in the pathogenesis and clinical outcome of OSCC. Genome-wide expression and methylation profile analysis revealed that genes such as ADHFE1 (alcohol dehydrogenase, iron containing 1) and ALDH1A2 (aldehyde dehydrogenase 1 family, member A2) were frequently hyper-methylated and downregulated in OSCC samples. Furthermore, the research group identified microRNAs viz., miR-30a and miR-379 which can influence the expression of *DNMT3B* that is involved in the process of methylation. Then it was shown that tobacco usage and betel quid chewing resulted in the dysregulation of microRNAs which ultimately resulted in the upregulation of *DNMT3B*. Chou et al., showed that arecoline, a betel-nut alkaloid recruited *DNMT3B *which in-turn regulates the *miR-486-3p/DDR1* genes. The upregulation of miR-486-3p exhibited a tumor suppressor activity by inhibiting tumor growth and inducing apoptosis, by targeting the 3’-UTR of Discoidin domain receptor-1 (DDR1). 

Computational approaches have been considered to be the boon for biologist. An exhaustive collection of data can be analyzed within a short time and cost-effective way. Several studies are emerging out in identifying novel genetic markers which can be further applied for population based screening (Vijayashree and Paramasivam, 2020; Anita et al., 2020). The limitations of the study were (a) the dataset selected had individuals from different ethnic groups or populations, which made data analysis and interpretation to be more generic rather than specific, (b) since both genetic and epigenetic factors act congruently to exhibit a disease phenotype, investigations on epigenetic factors such as influence of methylation, histone modification, microRNA interference could provide clues on trans-generational effects. Accumulation of knowledge on the methylation process, the identification of *DNMT* inhibitors have revolutionised the field of molecular medicine (Abreu et al., 2008). More details on the effect of variants especially the promoter and regulatory variants of the *DNMT* gene would open new avenues towards identification of prognostic and diagnostic markers. In conclusion, authors declare that advancements of computational approaches in biology have lead to the development of methods which can be used for identification of genetic abnormalities within a short span of time in a cost effective manner. The present study is one such attempt to accumulate information related to the genetic abnormalities in the *DNMT* and *HNSCC*. Findings of the study might aid in solving the puzzle underlying the epigenetic mechanisms and their role in *HNSCC.*
